# Elegant Medicines

**Published:** 1872-11

**Authors:** 


ELEGANT MEDICINES.—We have received from the house of Wm. R. Warner & Co., of Philadelphia, through Mr. C. M. Lyman, of this city, an elegant box containing specimens of the preparations of this firm. The medicines are in the form of sugar-coated pills and granules, and include the most common and useful medicines. They are put up in an elegant and, as far as we can ascertain, in a reliable form. Messrs. Warner & Co. are extensively engaged in the manufacture of sugar-coated pills and other pharmaceutical preparations, and are favorably known as dealers in pure and reliable medicines. We have on several occasions used their pills, and have found them to give good satisfaction. We are under many obligations to these gentlemen for the medicines which they have kindly left for our inspection, as well as for the elegant case in which they were contained. Mr. C. M. Lyman, No. 296 Main street, in this city, has Messrs. Warner & Co.'s goods for sale.

